# Complete genome sequences of *Acetobacterium wieringae* strains Y and N, capable of conjugated diene biohydrogenation

**DOI:** 10.1128/mra.00432-25

**Published:** 2025-09-03

**Authors:** Huijuan Jin, Hongyan Wang, Haiwei Wei, Jiaojiao Yang, Hengyi Liao, Xuhao Wang, Yiji Zhang, Xin Wang, Jingjing Wang, Siqi Huang, Shujing Yang, Xiuying Li, Jun Yan, Yi Yang

**Affiliations:** 1 Key Laboratory of Pollution Ecology and Environmental Engineering, Institute of Applied Ecology, Chinese Academy of Sciences 74763, Shenyang, Liaoning, China; 2 National Field Observation and Research Station of Shenyang Agro-ecosystems, Institute of Applied Ecology, Chinese Academy of Sciences 74763, Shenyang, Liaoning, China; 3 University of Chinese Academy of Sciences, Beijing, China; 4 School of Pharmaceutical Engineering, Shenyang Pharmaceutical University 58575, Shenyang, Liaoning, China; 5 Key Laboratory of Forest Ecology and Silviculture, Institute of Applied Ecology, Chinese Academy of Sciences 74763, Shenyang, Liaoning, China; University of Southern California, Los Angeles, California, USA

**Keywords:** *Acetobacterium*, isoprene, 1,3-butadiene, biohydrogenation, anaerobes, acetogenesis

## Abstract

We report the complete genome sequences of two acetogenic *Acetobacterium wieringae* strains, Y and N, isolated from river sediment. Under anaerobic acetogenic conditions, strain Y biohydrogenates isoprene, while strain N transforms 1,3-butadiene to 1-butene.

## ANNOUNCEMENT

*Acetobacterium wieringae* is a strictly anaerobic bacterium in the order *Eubacteriales* (1). Here, *A. wieringae* strains Y and N, capable of metabolizing isoprene and 1,3-butadiene—pollutants linked to ozone chemistry and carcinogenicity (2, 3)—were isolated from sediment collected from the Xi River, Shenyang, China (41.6628°N, 123.1055°E) in July 2017 (4, 5). Sediment samples were collected from the riverbed using alcohol-sterilized shovels, placed into 2-L serum bottles filled with river water to eliminate headspace, sealed, and transported to the laboratory on ice. Anaerobic microcosms were prepared by inoculating 2 g sediment into a bicarbonate-buffered mineral salt medium (6) containing lactate, hydrogen, and either isoprene or 1,3-butadiene, and incubated at 30°C for 2 weeks. To obtain pure colonies, enrichment cultures were serially diluted to extinction after successive transfers and plated on the same medium solidified with 1% low-melting-point agarose as previously described (6). 16S rRNA gene sequencing was performed using colony PCR with universal primers 27F/1492R following standard protocols (7), showing 99.7% identity to *A. wieringae* DSM 1911 (NR_026324).

Genomic DNA was extracted from 0.8 L stationary-phase cultures grown with either isoprene or 1,3-butadiene using the SDS method (8), and the same DNA extract was used for both PacBio and Illumina library preparation. For PacBio sequencing, DNA underwent ~10 kb BluePippin size selection and library preparation with the SMRTbell Template Prep Kit (Pacific Biosciences, Menlo Park, CA, USA) following the manufacturer’s protocol, then sequenced on the PacBio Sequel II (9). Illumina paired-end (2 × 150 bp) libraries were generated using the NEBNext Ultra DNA Library Prep Kit (New England Biolabs, Ipswich, MA, USA) according to the manufacturer’s instructions and sequenced on a NovaSeq 6000 using an S4 flow cell. Raw reads were quality-trimmed using Trimmomatic v0.39 and assessed using FastQC v0.11.9 (10, 11), yielding 8,379,940 Illumina reads (Q30 = 93.46%) and 261,179 PacBio reads (*N_50_* = 14,167 bp) for strain Y, and 7,444,304 Illumina reads (Q30 = 93.24%) and 105,647 PacBio reads (*N_50_* = 12,924 bp) for strain N. Illumina and PacBio reads were co-assembled with Unicycler v0.50 (12), and the quality of the assemblies was evaluated using CheckM v1.2.2 (13). Annotation was conducted via NCBI Prokaryotic Genome Annotation Pipeline v6.7. Digital DNA-DNA hybridization (dDDH) and average nucleotide identity (ANI) were analyzed using TYGS (https://tygs.dsmz.de/) and JSpeciesWS v4.1.1, respectively (14). Default parameters were used for all software.

While CheckM estimated completeness for strains Y and N at 98.57% and 99.29%, respectively (both with 0.71% contamination), their circular, gapless assemblies with full rRNA operons indicate complete genomes. Strains Y and N possess circular chromosomes of 4,082,090 bp and 4,094,057 bp, encoding 3,846 and 3,874 genes, respectively, each with 16 rRNAs, 58 tRNAs, and four ncRNAs, and lacking plasmids. Both genomes have a GC content of 44.2% and share 73.7% dDDH and 97.5% ANI with *A. wieringae* DSM 1911 (GCA_001766835). Notable genes include those encoding the complete Wood-Ljungdahl pathway and putative diene reductase cluster comprising a key FAD-dependent oxidoreductase (LNN31_08025 in strain Y and OU988_08160 in strain N), three pleiotropic nickel chaperones, and a 4Fe-4S binding protein.

## Data Availability

The genomes of strains Y and N are available at the DNA Data Bank of Japan, the European Nucleotide Archive, and GenBank under accession number CP087994 and CP113903. The BioSample and BioProject accession numbers are SAMN23007773 and PRJNA778957 for strain Y and SAMN31888781 and PRJNA906009 for strain N. Raw reads were deposited in the Sequence Read Archive (SRA) under accession numbers SRR18091988 and SRR31728078 (PacBio) and SRR18087747 and SRR31728519 (Illumina).
